# Risk of severe infections in patients with triple-negative breast cancer treated with atezolizumab plus nab-paclitaxel: a real-world, postmarketing database study in Japan

**DOI:** 10.1007/s10147-025-02904-0

**Published:** 2025-10-30

**Authors:** Akinori Yuri, Sayuri Nakane, Yuki Miyano, Kana Yamada, Hiroshi Sugano, Erika Nakatsuji, Masahiko Aoki, Ayako Murayama

**Affiliations:** 1 Safety Science 2 Department, Drug Safety Division, Chugai Pharmaceutical Co., Ltd., 1-1 Nihonbashi-Muromachi 2-Chome, Chuo-Ku, Tokyo, 103-8324 Japan; 2 Safety Science 1 Department, Drug Safety Division, Chugai Pharmaceutical Co., Ltd, Tokyo, Japan

**Keywords:** Atezolizumab, Infection, Postmarketing surveillance, Propensity score matching, Real-world data, Triple-negative breast cancer

## Abstract

**Background:**

An increased risk of infection has been suggested for patients with triple-negative breast cancer (TNBC) treated with atezolizumab plus nab-paclitaxel based on the IMpassion130 trial.

**Methods:**

This Japanese postmarketing study aimed to compare the incidence of severe infections in patients with TNBC treated with atezolizumab plus nab-paclitaxel versus nab-paclitaxel alone. Anonymized patient records regarding medical claims and laboratory tests were extracted from the Japanese Medical Data Vision database. Patients with a drug index date of November 27, 2019,–May 31, 2022, were included in the analysis. Based on the published literature, severe infections were declared when a patient had a confirmed diagnosis of infection, a hospitalization record, infection as the primary reason for hospitalization, and a record of immunological infection tests. In the sensitivity analysis, the definition of severe infections was modified as a combination of confirmed infection diagnosis and intravenous antibacterial drug prescription records.

**Results:**

Overall, 321 and 319 patients were included in the exposure (atezolizumab plus nab-paclitaxel) and control (nab-paclitaxel alone) groups, respectively. After adjusting for standardized mortality/morbidity ratio weighting, the baseline characteristics were balanced between the groups. The incidence rate ratio (exposure/control) of severe infections was estimated at 3.29 (95% confidence interval [CI]: 0.93–13.53) originally and 1.05 (95% CI: 0.56–1.84) in the sensitivity analysis. Additional analyses supported the appropriateness of the revised definition of severe infections.

**Conclusions:**

Overall, our results did not indicate a significant increase in the risk of severe infections with atezolizumab plus nab-paclitaxel in daily clinical practice. Further research is required.

**Supplementary Information:**

The online version contains supplementary material available at 10.1007/s10147-025-02904-0.

## Introduction

Breast cancer is the most common cancer in women worldwide [1]. Triple-negative breast cancer (TNBC) is characterized by the absence of the expression of hormone receptors and human epidermal growth factor receptor 2 (HER2) on the tumor cell surface [2] and accounts for 10–15% of breast cancer cases [3, 4]. TNBC is associated with a poor prognosis, as illustrated by an estimated median overall survival of 9.3 to 13.7 months [5].

Immune checkpoint inhibitors in combination with chemotherapy have emerged as an effective option for the treatment of TNBC, and the benefit is pronounced in patients who test positive for programmed death ligand 1 (PD-L1) [6]. Atezolizumab is an immunoglobulin G1 monoclonal antibody that selectively inhibits the interaction of PD-L1 with the cell-surface proteins PD-1 and B7-1 [7]. Atezolizumab (840 mg intravenous infusion) plus nab-paclitaxel was approved for PD-L1-positive, unresectable, or recurrent TNBC in Japan in 2019 [8] based on the IMpassion130 trial [7, 9]. This combination regimen is strongly recommended in the 2022 Japanese breast cancer guidelines [10]. Atezolizumab plus nab-paclitaxel has also been approved in the European Union for the treatment of PD-L1-positive, unresectable, locally advanced or metastatic TNBC [11].

Clinical trial results have raised concerns regarding an increased risk of infections in patients with TNBC treated with atezolizumab plus nab-paclitaxel [7, 9]. In the IMpassion130 trial, the incidence rates of urinary tract infection (11.7% vs 10.5%) and upper respiratory tract infection (10.6% vs 9.1%) were slightly higher with atezolizumab plus nab-paclitaxel than with nab-paclitaxel alone [7]. A subgroup analysis of IMpassion130, which included 65 Japanese patients with advanced TNBC, also indicated a higher incidence of urinary tract infections with atezolizumab plus nab-paclitaxel than with nab-paclitaxel alone (11.8% vs 3.3%) [9]. Real-world evidence on the risk of severe infections in patients treated with this regimen is warranted.

This postmarketing database study was conducted as part of the pharmacovigilance surveillance activities specified in the Japanese Risk Management Plan for atezolizumab [12]. We previously reported the impact of atezolizumab on the onset of febrile neutropenia in patients with non-small cell lung cancer (NSCLC) receiving chemotherapy using a Japanese healthcare database [13]. The current study aimed to compare the incidence of severe infections in patients with TNBC treated with atezolizumab plus nab-paclitaxel versus nab-paclitaxel alone using a similar study design.

## Patients and methods

### Study design and data source

This study employed a retrospective cohort design and used anonymized patient information extracted from the Medical Data Vision (MDV) database of Japan. The MDV database contains medical claims and laboratory test data derived from 519 Japanese acute-care hospitals (data as of August 2024) [14]. The study period (data period) was set as January 1, 2012, to December 31, 2022. Patients chosen from the database were categorized into the exposure (atezolizumab plus nab-paclitaxel) or control (nab-paclitaxel alone) group based on the study regimen received. The index date was defined as the date of the first atezolizumab or nab-paclitaxel dose, whichever occurred earlier. Patient data were observed from the date of the first administration of the study regimen until (1) 210 days after the index date, (2) date of onset of severe infections, (3) end of the study regimen, (4) day before prescription of atezolizumab 1200 mg intravenous infusion (dose approved for indications other than TNBC, such as NSCLC, suggesting a potential change in the primary disease being treated), (5) day before switching to another study regimen, or (6) day before prescription of other anticancer drugs, whichever occurred first. The period of 210 days after the index date was set to capture >90% of outcomes of interest based on a feasibility study that estimated 205 days as a period required to capture 90% of outcomes of interest (Chugai Pharmaceutical Co., Ltd., data on file).

### Patient inclusion and exclusion criteria

Patients with a confirmed diagnosis of breast cancer based on the International Classification of Diseases, 10th Revision codes (Supplementary Table 1) during the study period were eligible for inclusion. Patients who were newly initiated on atezolizumab plus nab-paclitaxel or nab-paclitaxel alone in the month of breast cancer diagnosis (index date: November 27, 2019 [date of listing of atezolizumab on the National Health Insurance drug price list], to May 31, 2022) were included in the analysis. Patients were excluded if they had a short-term (≤89 days) data record before the index date without a pathological diagnosis, history of study regimen treatment, severe infections within 30 days before the index date, HER2-positive breast cancer (Supplementary Table 1), history of adjuvant or neoadjuvant chemotherapy, history of other cancer regimens, or an index date equal to the last visit.

### Definition of severe infections

In this study, an algorithm validated for the MDV database [15] was used to identify severe infections. Severe infections were declared when all the following criteria were met for the MDV database record in a single patient: (1) confirmed diagnosis of infection (excluding suspected diagnoses), (2) hospitalization record, (3) confirmed diagnosis of infection as a primary reason for hospitalization, and (4) record of immunological infection tests (Supplementary Table 2) performed between the month before the admission date and the discharge date (original definition; Supplementary Fig. 1). The date of the onset of severe infection was defined as the date of admission.

### Propensity score matching

Propensity score matching using standardized mortality/morbidity ratio weighting (SMRW) was applied to match patient characteristics between the groups. To calculate propensity scores, the following covariates were selected by referring to published literature [16, 17]: age (years), duration of breast cancer (months), coronavirus disease 2019 (COVID-19) season, presence or absence of renal impairment, presence or absence of hepatic impairment, presence or absence of lymph node metastasis, presence or absence of diabetes, Charlson Comorbidity Index (CCI) score, history of surgery for breast cancer, history of non-breast cancer surgery, history of radiotherapy, history of anticancer drug administration, and presence or absence of steroid use.

### Outcomes of interest

Primary analysis was performed to estimate the incidence rate ratio (IRR) of severe infections (exposure/control group) in the unadjusted and SMRW-adjusted populations. The time to onset of severe infection was analyzed in the exposure and control groups.

### Sensitivity analyses

Sensitivity analyses were performed to evaluate the robustness of the primary analysis. In the current breast cancer population, hospitalizations due to noninfectious diseases may have occurred more frequently than in a previous validation study of the severe infection algorithm [15]. Patients included in the current postmarketing study may have undergone infection tests as part of the prehospitalization examinations, even if they had no signs or symptoms of infections (Supplementary Fig. 1). Although the original definition of severe infections was based on a validated algorithm [15], these circumstances may have affected the results of the current study for TNBC.

The original definition of severe infections was modified to generate Outcome Definitions 1–3, as summarized in Supplementary Fig. 1. Outcome Definition 1 was generated by excluding immunological infection tests from the definition and adding the record of prescription of intravenous antibacterial drugs (injections or infusions) listed in Supplementary Table 3. In Outcome Definition 2, the definition was further modified as a combination of a confirmed diagnosis of infection and intravenous antibacterial drug prescription records to broaden the definition of infections. Hospitalization was excluded from Outcome Definition 2 based on the hypothesis that the definition used in the previous validation study [15] was unable to capture infections that occurred during hospitalization; thus, it may not have produced sufficient sensitivity in our study, which likely included a high number of hospitalized patients. Outcome Definition 3 was generated by excluding COVID-19-related diagnoses from the original definition of severe infections. Starting in 2020, after the regulatory approval of atezolizumab for TNBC in 2019, Japan experienced the COVID-19 pandemic [18]. This unprecedented circumstance may have affected the trend of severe infections (Supplementary Fig. 1).

In addition, propensity score trimming was applied to mitigate the effects of residual confounding factors [19]. The minimum and maximum propensity scores were calculated for each group. Patients with propensity scores not within the score range of another group were excluded from each group.

### Statistical analyses

The sample size was initially set at 250 patients per group based on the incidence of severe infections in the control group estimated from the IMpassion130 trial (16.1 per 100 person-years), an observation period of 16.1 weeks, and an assumed twofold higher incidence of severe infections in the exposure group than in the control group. This sample size was expected to determine the IRR of severe infections with a 95% confidence interval (CI) of 1.01–3.95. All patients available at the time of analysis were included to increase the statistical power.

The incidence rates of severe infections (per person-years), IRRs, and 95% CIs were estimated for the unadjusted and SMRW-adjusted populations. The time to severe infection onset was estimated using the Kaplan–Meier method. All analyses were performed using SAS Version 9.4 (SAS Institute, Inc., Cary, NC, USA).

### Additional *post hoc* analyses

While propensity score weighting was applied, additional *post hoc* analyses were performed. An additional analysis was performed to evaluate whether the incidence of severe infections was influenced by differences in the occurrence of clinical events between the exposure and control groups. A descriptive analysis was performed on the occurrence of clinical events related to severe infections (hospitalization, surgery, radiotherapy, and steroid use) during the observation period using the original definition.

As recommended in the Japanese guidelines for postoperative use of antibacterials, it is possible that antibacterial drug prescriptions for 48–72 h were for the prophylaxis of postoperative infections [20]. Another additional analysis evaluated whether antibacterial drugs were prescribed for prophylactic purposes, thus affecting the results of Outcome Definition 2. Antibacterial drug prescription patterns (name of the prescribed antibacterial drugs and treatment duration) and patient inpatient/outpatient status at the time of antibacterial prescription for severe infection onset were analyzed for Outcome Definition 2.

## Results

### Patient disposition

**Fig. 1:**
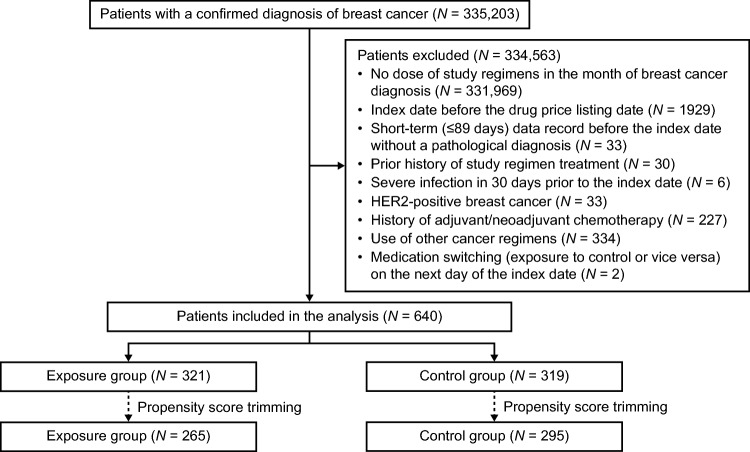
Patient disposition. Exposure group: atezolizumab plus nab-paclitaxel. Control group: nab-paclitaxel alone. *HER2* Human epidermal growth factor receptor 2

Among the 335,203 patients with a confirmed diagnosis of breast cancer in the MDV database, 321 and 319 were included in the exposure and control groups, respectively (Fig. 1). After propensity score trimming, 265 and 295 patients were included in the exposure and control groups, respectively (Fig. 1).

### Baseline characteristics

Before SMRW adjustment, the mean ± standard deviation (SD) age was 59.2 ± 12.6 years in the exposure group and 59.2 ± 12.7 years in the control group, and women accounted for 99.7% and 100.0% of patients in the exposure and control groups, respectively (Table 1). After adjusting for SMRW, the baseline characteristics were well balanced between the groups (Supplementary Fig. 2a, b, Table 1, Supplementary Table 4). The standardized difference for the mean ± SD CCI score was 0.535 before adjustment and −0.090 after SMRW adjustment. The standardized difference for steroid use was −0.323 and −0.095 before and after SMRW adjustment, respectively (Table 1, Supplementary Table 4).

**Table 1:** Baseline characteristics

	Unadjusted			Adjusted by SMRW		
	Exposure group (*N* = 321)	Control group (*N* = 319)	Standardized difference	Exposure group (*N* = 321.0)	Control group (*N* = 298.3)	Standardized difference
Female sex	320 (99.7)	319 (100.0)	−0.079	320.0 (99.7)	298.3 (100.0)	−0.079
Age (years)	59.2 ± 12.6	59.2 ± 12.7	−0.001	59.2 ± 12.6	61.1 ± 11.2	−0.162
Breast cancer duration (months)	27.58 ± 24.58	22.18 ± 28.76	0.202	27.58 ± 24.58	33.54 ± 28.74	−0.223
Renal impairment	31 (9.7)	37 (11.6)	−0.063	31.0 (9.7)	26.6 (8.9)	0.025
Hepatic impairment	100 (31.2)	91 (28.5)	0.057	100.0 (31.2)	98.6 (33.1)	−0.041
Lymph node metastasis	167 (52.0)	86 (27.0)	0.530	167.0 (52.0)	134.5 (45.1)	0.139
Diabetes	45 (14.0)	49 (15.4)	−0.038	45.0 (14.0)	40.1 (13.4)	0.017
CCI score	7.5 ± 2.8	5.8 ± 3.7	0.535	7.5 ± 2.8	7.8 ± 2.8	−0.090
Prior breast cancer surgery	6 (1.9)	20 (6.3)	−0.224	6.0 (1.9)	4.2 (1.4)	0.037
Prior non-breast cancer surgery	193 (60.1)	158 (49.5)	0.214	193.0 (60.1)	182.7 (61.3)	−0.023
Prior radiotherapy	135 (42.1)	48 (15.0)	0.627	135.0 (42.1)	102.6 (34.4)	0.158
Prior anticancer treatment	268 (83.5)	240 (75.2)	0.205	268.0 (83.5)	251.5 (84.3)	−0.023
Steroid use	129 (40.2)	179 (56.1)	−0.323	129.0 (40.2)	133.8 (44.9)	−0.095

Exposure group: atezolizumab plus nab-paclitaxel. Control group: nab-paclitaxel alone. Data are presented as *n* (%) or mean ± SD unless otherwise specified

*CCI* Charlson Comorbidity Index, *SD* Standard deviation, *SMRW* Standardized mortality/morbidity ratio weighting

### Incidence of severe infections and IRRs: primary and sensitivity analyses

Using the original definition of severe infections, the incidence rate of severe infections adjusted by the SMRW was 0.11 (95% CI: 0.04–0.17) per person-years in the exposure group and 0.03 (95% CI: −0.01 to 0.07) per person-years in the control group. The IRR of severe infections was estimated at 3.29 (95% CI: 0.93–13.53; Table 2). Similar results were obtained in the sensitivity analyses when Outcome Definitions 1 and 3 were used. The use of Outcome Definition 2, when adjusted by SMRW, resulted in incidence rates of 0.52 (95% CI: 0.38–0.66) per person-years in the exposure group and 0.49 (95% CI: 0.33–0.65) per person-years in the control group. The IRR was 1.05 (95% CI: 0.56–1.84), indicating no significant difference in the incidence of severe infections between the groups (Table 2). The incidence of severe infections and IRRs after propensity score trimming did not differ substantially from those obtained from analyses using the original definition (Table 2).

**Table 2:** Incidence of severe infections (per person-years) and IRRs: primary and sensitivity analyses

			Unadjusted		Adjusted by SMRW	
			Value	95% CI	Value	95% CI
Original definition	Incidence	Exposure	0.11	0.04–0.17	0.11	0.04–0.17
		Control	0.03	−0.01 to 0.06	0.03	−0.01 to 0.07
	IRR	Exposure/control	4.14	0.92–18.66	3.29	0.93–13.53
Outcome Definition 1	Incidence	Exposure	0.11	0.04–0.17	0.11	0.04–0.17
		Control	0.01	−0.01 to 0.04	0.02	−0.01 to 0.06
	IRR	Exposure/control	8.28	1.07–64.11	4.85	1.07–7.76
Outcome Definition 2	Incidence	Exposure	0.52	0.38–0.66	0.52	0.38–0.66
		Control	0.34	0.21–0.48	0.49	0.33–0.65
	IRR	Exposure/control	1.51	0.94–2.41	1.05	0.56–1.84
Outcome Definition 3	Incidence	Exposure	0.11	0.04–0.17	0.11	0.04–0.17
		Control	0.03	−0.01 to 0.06	0.03	−0.01 to 0.07
	IRR	Exposure/control	4.14	0.92–18.66	3.29	0.93–13.53
Propensity score trimming	Incidence	Exposure	NA	NA	0.10	0.04–0.17
		Control	NA	NA	0.03	−0.01 to 0.07
	IRR	Exposure/control	NA	NA	3.19	0.81–15.19

Exposure group: atezolizumab plus nab-paclitaxel, Control group: nab-paclitaxel alone

*CI* Confidence interval, *IRR* Incidence rate ratio, *NA* Not applicable, *SMRW* Standardized mortality/morbidity ratio weighting

### Time to onset of severe infections

Survival analysis using the original definition did not indicate a substantial difference in the time to severe infection onset between the exposure and control groups (Supplementary Fig. 3a). Similarly, no substantial difference was observed between the groups in the sensitivity analysis using Outcome Definition 2 (Supplementary Fig. 3b) or analyses adjusted for SMRW (Supplementary Fig. 4a, b).

### Occurrence of clinical events related to severe infection: additional analyses

After adjustment by SMRW and using the original definition, the number of hospitalizations during the observation period was numerically higher in the exposure group than in the control group (mean ± SD, 0.6 ± 1.2 vs 0.4 ± 0.8; Supplementary Table 5). Steroid use was less common in the exposure group than in the control group (63.2% vs 77.6%; Supplementary Table 5).

### Antibacterial drug use: additional analyses

The most common antibacterial intravenous injections or infusions prescribed to both groups are shown in Supplementary Table 6. When using Outcome Definition 2, the median duration of antibacterial drug prescription was 7.0 days in the exposure group and 7.5 days in the control group (Table 3). In both groups, a vast majority (>90%) of the patients were prescribed antibacterial drugs in inpatient settings (Table 3).

**Table 3:** Prescription of antibacterial drugs during the observation period (Outcome Definition 2): additional analysis

	Exposure group (*N* = 51)	Control group (*N* = 26)
Duration of antibacterial drug prescription for severe infection onset (days)		
Mean ± SD	7.47 ± 4.71	7.88 ± 3.93
Median (range)	7.00 (2.0–25.0)	7.50 (3.0–17.0)
Treatment setting at the time of antibacterial drug prescription for severe infection onset, *n* (%)		
Outpatient	2 (3.9)	2 (7.7)
Inpatient	49 (96.1)	24 (92.3)

Exposure group: atezolizumab plus nab-paclitaxel, Control group: nab-paclitaxel alone

*SD* Standard deviation

## Discussion

In patients with TNBC, the IMpassion130 trial suggested an increased risk of severe infections with atezolizumab plus nab-paclitaxel [7, 9]. This Japanese postmarketing database study assessed the risk of severe infections in patients with TNBC treated with this combination regimen in a real-world setting.

The primary analysis using the original definition suggested a trend toward an increased risk of severe infections with atezolizumab plus nab-paclitaxel (IRR: 3.29, 95% CI: 0.93–13.53). However, the estimated incidence rates (0.11 and 0.03 per person-years in the exposure and control groups, respectively) were lower than that anticipated from the IMpassion130 trial (0.16 per person-years in the control group). The current database study did not exclude patients with poor performance status; thus, it is less likely that the true incidence rate of severe infections is lower than that observed in the IMpassion130 trial, which included only patients with an Eastern Cooperative Oncology Group performance status of 0 or 1 [7]. When the original definition was used, 184 and 123 hospitalizations were observed in the exposure and control groups, respectively (data not shown). This imbalance in the number of hospitalization events indicates that the sensitivity of the primary analysis may have been compromised owing to the inclusion of hospitalization in the definition of severe infections. Although the details of hospitalizations in the validation study [15] are not clear, a majority of the hospitalizations in our study can be attributed to reasons other than severe infections. This difference in hospitalization events may have affected the sensitivity and positive predictive value of the original outcome definition.

The sensitivity analysis using Outcome Definition 2 showed similar incidence rates between the groups (0.52 and 0.49 per person-years in the exposure and control groups, respectively; IRR: 1.05, 95% CI: 0.56–1.84). Outcome Definition 2 was designed to broaden the definition of severe infections by excluding hospitalization and immunological infection test records and including the prescription of intravenous antibacterial drugs. The use of intravenous antibacterials is aligned with the Grade 3 infection defined in the Common Terminology Criteria for Adverse Events [21]. The mean treatment duration (approximately 7–8 days) and high proportion of patients treated with intravenous antibacterial drugs in inpatient settings (>90%) indicated that antibacterials were most likely prescribed for the treatment of infections rather than for prophylactic purposes. Thus, this definition was deemed suitable for capturing the onset of severe infections in real-world scenarios.

Overall, a comparison of the results of the primary and sensitivity analyses indicated that the difference in the IRR may be attributed to the difference in the definition of severe infections. The original definition [15] may not be able to capture certain severe infection events, such as infections that required hospitalization but were not the primary reason and those that developed during hospitalization. Accordingly, the incidence rates of severe infections may have been underestimated in the primary analysis. The findings of other sensitivity analyses were consistent with the data of the primary analysis, which indicated that replacing immunological infection tests with intravenous antibacterial drug prescriptions (Outcome Definition 1), exclusion of COVID-19-related diagnoses (Outcome Definition 3), or propensity score trimming had little effect on the results.

Taken together, our study indicates no significant increase in the risk of severe infections with atezolizumab plus nab-paclitaxel, although this is specified as an important potential risk in the Japanese Risk Management Plan for atezolizumab [12]. However, clinicians should remain vigilant and continue to monitor patients for potential infections per the standard practice in cancer chemotherapy. Capturing data on severe infections using spontaneous adverse event reporting systems is necessary as part of pharmacovigilance activities [22, 23].

The strengths of this study include the use of a large real-world cohort, a validated algorithm to define severe infections [15], and propensity score matching to control for confounding factors. However, this study has some limitations. First, the findings may not be generalizable to regions other than Japan because the definition of severe infections used in this study [15] may not be applicable to other countries. Second, the use of the MDV database limited the patients to those treated at acute-care hospitals in Japan, and the clinical data available from the database were limited. Third, our results may have been biased owing to several factors, including antibiotic use for noninfectious events (e.g., mild pneumonitis as immune-related adverse events), leading to overestimated infection rates in both groups, especially in the exposure group. Furthermore, the COVID-19 pandemic may have influenced patients’ healthcare-seeking behaviors, diagnostic practices, and the overall incidence of respiratory infections in ways that could not be fully adjusted for. Lastly, other inherent limitations of the study, such as potential residual confounding and possible misclassification of infections, may have affected the interpretation of the study.

In conclusion, this real-world study of Japanese patients with TNBC did not indicate a significant increase in the risk of severe infections with atezolizumab plus nab-paclitaxel compared with nab-paclitaxel alone. Continued infection monitoring through spontaneous adverse event reporting and further research are warranted to better understand the potential risk of infection associated with this combination therapy in routine clinical practice.

## Supplementary Information

Below is the link to the electronic supplementary material.

Supplementary file1 (DOCX 1487 KB)

## Data Availability

All data supporting the findings of this study are available within the paper and its Supplementary Information.
