# Supplementary material for: Risk of severe infections in patients with triple-negative breast cancer treated with atezolizumab plus nab-paclitaxel: a real-world, postmarketing database study in Japan
Source: Int J Clin Oncol. 2025 Oct 30;30(12):2541–8. doi: 10.1007/s10147-025-02904-0 (PMC12644192; doi:10.1007/s10147-025-02904-0)
Supplement: Supplementary file 1 — Supplementary file1 (DOCX 1487 KB) [file 10147_2025_2904_MOESM1_ESM.docx]

**Supplementary materials**

**Article title**

Risk of severe infections in patients with triple-negative breast cancer treated with atezolizumab plus nab-paclitaxel: a real-world, postmarketing database study in Japan

**Journal name**

*International Journal of Clinical Oncology*

**Author names**

Akinori Yuri, Sayuri Nakane, Yuki Miyano, Kana Yamada, Hiroshi Sugano, Erika Nakatsuji, Masahiko Aoki, Ayako Murayama

**Affiliation and e-mail address of the corresponding author**

Safety Science 2 Department, Drug Safety Division, Chugai Pharmaceutical Co., Ltd.

E-mail address: [yuri.akinori58@chugai-pharm.co.jp](mailto:yuri.akinori58@chugai-pharm.co.jp)

**Table of contents**

**Supplementary Table 1**. ICD-10 code list for breast cancer

**Supplementary Table 2**. List of immunological infection tests

**Supplementary Table 3**. List of antibacterials

**Supplementary Table 4**. Baseline characteristics (full data)

**Supplementary Table 5**. Clinical events related to severe infections during the observation period (original definition)

**Supplementary Table 6**. Most commonly prescribed antibacterial drugs (Outcome Definition 2): additional analysis

**Supplementary Fig. 1**. Definitions of severe infections

**Supplementary Fig. 2**. Propensity score distribution (a) before and (b) after weighting

**Supplementary Fig. 3**. Time to the onset of severe infections using (a) original definition and (b) Outcome Definition 2 (sensitivity analysis)

**Supplementary Fig. 4**. Time to the onset of severe infections using (a) Outcome Definition 1 (adjusted for SMRW) and (b) Outcome Definition 3 (adjusted for SMRW)

**Supplementary Table 1**. ICD-10 code list for breast cancer

| ICD-10 code | Disease code | Disease name |
| --- | --- | --- |
| Breast cancer (inclusion criteria) | | |
| C500 | 8838489 | Mammary Paget’s disease |
| C500 | 8845450 | Nipple breast cancer |
| C500 | 8845452 | Areolar breast cancer |
| C501 | 8838483 | Central breast cancer |
| C502 | 8838476 | Upper-inner quadrant breast cancer |
| C503 | 8838465 | Lower-inner quadrant breast cancer |
| C504 | 8838475 | Upper-outer quadrant breast cancer |
| C505 | 8838464 | Lower-outer quadrant breast cancer |
| C506 | 8845449 | Mammary gland axillary-tail breast cancer |
| C506 | 8848843 | Axillary breast cancer |
| C508 | 8845451 | Malignant tumor of overlapping sites of breast |
| C508 | 8848690 | Ectopic breast cancer |
| C509 | 1749004 | Postoperative breast cancer |
| C509 | 1749008 | Breast cancer |
| C509 | 1749009 | Breast cancer recurrence |
| C509 | 1749011 | Malignant mammary gland tumor |
| C509 | 1749015 | Malignant breast tumor |
| C509 | 1749017 | Breast sarcoma |
| C509 | 8830917 | Inflammatory breast cancer |
| C509 | 8842665 | Malignant phyllodes tumor |
| C509 | 8842759 | Advanced breast cancer |
| C509 | 8845025 | Breast liposarcoma |
| C509 | 8848646 | Breast angiosarcoma |
| C509 | 8848647 | Breast fibrosarcoma |
| C509 | 8848722 | Invasive ductal carcinoma |
| C509 | 8848743 | Multiple breast cancer |
| C509 | 8848773 | Papillotubular carcinoma |
| C509 | 8849183 | Mammary gland scirrhous carcinoma |
| C509 | 8849184 | Mammary gland solid-tubular carcinoma |
| C509 | 8849815 | Breast cancer local recurrence |
| C509 | 8849816 | Breast cancer postoperative recurrence on chest wall |
| HER2-positive breast cancer (exclusion criteria) | | |
| C509 | 8844799 | Breast cancer - HER2 overexpression |
| C509 | 8849699 | HER2 positive breast cancer |

*HER2* human epidermal growth factor receptor 2, *ICD-10* International Classification of Diseases, 10th Revision

**Supplementary Table 2**. List of immunological infection tests

| Category | Medical fee receipt code | Medical fee receipt name |
| --- | --- | --- |
| D012 | 160007110 | Clostridium difficile antigen qualitative |
| D012 | 160039810 | STS qualitative |
| D012 | 160039910 | ASO qualitative |
| D012 | 160040110 | Toxoplasma antibody semi quantitative |
| D012 | 160040710 | STS quantitative |
| D012 | 160040810 | ASK qualitative |
| D012 | 160040910 | Treponema pallidum antibody qualitative |
| D012 | 160041010 | Mycoplasma antibody qualitative |
| D012 | 160041510 | Treponema pallidum antibody quantitative |
| D012 | 160041610 | Adenoviral antibody titer (qualitative/semi quantitative/quantitative) |
| D012 | 160041710 | Coxsackie virus antibody titer (qualitative/semi quantitative/quantitative) |
| D012 | 160041810 | Cytomegalovirus antibody titer (qualitative/semi quantitative/quantitative) |
| D012 | 160041910 | EB virus antibody titer (qualitative/semi quantitative/quantitative) |
| D012 | 160042010 | Echovirus antibody titer (qualitative/semi quantitative/quantitative) |
| D012 | 160042110 | Herpes virus antibody titer (qualitative/semi quantitative/quantitative) |
| D012 | 160042210 | Influenza virus type A antibody titer (qualitative/semi quantitative/quantitative) |
| D012 | 160042310 | Influenza virus type B antibody titer (qualitative/semi quantitative/quantitative) |
| D012 | 160042410 | Mumps virus antibody titer (qualitative/semi quantitative/quantitative) |
| D012 | 160042510 | Parainfluenza virus type 1 antibody titer (qualitative/semi quantitative/quantitative) |
| D012 | 160042610 | Parainfluenza virus type 2 antibody titer (qualitative/semi quantitative/quantitative) |
| D012 | 160042710 | Parainfluenza virus type 3 antibody titer (qualitative/semi quantitative/quantitative) |
| D012 | 160042810 | Poliovirus type 1 antibody titer (qualitative/semi quantitative/quantitative) |
| D012 | 160042910 | Poliovirus type 2 antibody titer (qualitative/semi quantitative/quantitative) |
| D012 | 160043010 | Poliovirus type 3 antibody titer (qualitative/semi quantitative/quantitative) |
| D012 | 160043110 | RS virus antibody titer (qualitative/semi quantitative/quantitative) |
| D012 | 160043210 | Rubella virus antibody titer (qualitative/semi quantitative/quantitative) |
| D012 | 160043310 | Measles virus antibody titer (qualitative/semi quantitative/quantitative) |
| D012 | 160043410 | Japanese Encephalitis virus antibody titer (qualitative/semi quantitative/quantitative) |
| D012 | 160043510 | Chlamydia psittaci antibody titer (qualitative/semi quantitative/quantitative) |
| D012 | 160043710 | Toxoplasma antibody |
| D012 | 160043810 | HTLV-1 antibody semi quantitative |
| D012 | 160044010 | Bordetella pertussis antibody semi quantitative |
| D012 | 160044110 | A group beta hemolytic streptococcus quick test qualitative |
| D012 | 160044210 | Herpes simplex virus antigen qualitative |
| D012 | 160044710 | FTA-ABS test qualitative |
| D012 | 160045210 | HTLV-1 antibody |
| D012 | 160046010 | Virus antibody titer (herpes) by globulin class |
| D012 | 160046210 | Virus antibody titer (rubella) by globulin class |
| D012 | 160058550 | Gonococcus antigen qualitative |
| D012 | 160104150 | Streptococcus pneumoniae antigen qualitative (cerebrospinal fluid) |
| D012 | 160104250 | Streptococcus pneumoniae antigen qualitative (urine) |
| D012 | 160109410 | Virus antibody titer (cytomegalovirus) by globulin class |
| D012 | 160112810 | Adenoviral antigen qualitative (stool) |
| D012 | 160112910 | Rotaviral antigen qualitative (stool) |
| D012 | 160115510 | Endotoxin |
| D012 | 160117510 | HIV-1 antibody |
| D012 | 160117610 | RS virus antigen qualitative |
| D012 | 160117710 | Tsutsugamushi antibody qualitative |
| D012 | 160117810 | Chlamydia trachomatis antibody by globulin class |
| D012 | 160117910 | Entamoeba histolytica antibody semi quantitative |
| D012 | 160118010 | Virus antibody titer by globulin class |
| D012 | 160118110 | HIV-1 antibody (Western blotting) |
| D012 | 160120610 | Chlamydia trachomatis antigen qualitative |
| D012 | 160121450 | Cytomegalovirus antibody |
| D012 | 160121510 | Virus antibody titer (EB) by globulin class |
| D012 | 160122150 | Hib antigen qualitative (spinal fluid) |
| D012 | 160125850 | Chickenpox virus antigen qualitative (epithelium cell) |
| D012 | 160141850 | Candida antigen qualitative |
| D012 | 160142650 | HTLV-1 antibody (Western blotting and line blotting) |
| D012 | 160148810 | Adenoviral antigen qualitative (except stool) |
| D012 | 160151350 | Toxoplasma IgM antibody |
| D012 | 160151950 | Cryptococcus antigen qualitative |
| D012 | 160152450 | Bordetella pertussis antibody |
| D012 | 160153050 | HIV-1 and -2 antibody qualitative |
| D012 | 160153150 | Mycoplasma antigen qualitative (FA method) |
| D012 | 160154450 | HIV-2 antibody (Western blotting) |
| D012 | 160157210 | Virus antibody titer (measles) by globulin class |
| D012 | 160157310 | Virus antibody titer (mumps) by globulin class |
| D012 | 160158250 | Anisakis IgG/IgA antibody |
| D012 | 160158350 | Aspergillus antigen |
| D012 | 160160150 | (1 -> 3)-beta-D-glucan |
| D012 | 160162750 | Escherichia coli, by serogroup |
| D012 | 160163850 | Cytomegalovirus pp65 antigen qualitative |
| D012 | 160164350 | E. coli O157 antigen, qualitative |
| D012 | 160167350 | Chlamydophila pneumoniae IgG antibody |
| D012 | 160167450 | Chlamydophila pneumoniae IgA antibody |
| D012 | 160167550 | Virus antibody titer by globulin class (human parvovirus B19) |
| D012 | 160168250 | E. coli O157 antibody, qualitative |
| D012 | 160169450 | Influenza virus antigen qualitative |
| D012 | 160172450 | Helicobacter pylori antibody qualitative/semiquantitative |
| D012 | 160172550 | Helicobacter pylori antibody |
| D012 | 160172750 | Rapid urease test qualitative |
| D012 | 160173450 | Mycobacterium tuberculosis complex antigen, qualitative |
| D012 | 160173650 | Mycobacterial antibody qualitative |
| D012 | 160174210 | Herpes simplex virus antibody titer (qualitative/semi quantitative/quantitative) |
| D012 | 160174310 | Varicella-zoster virus antibody titer (qualitative/semi quantitative/quantitative) |
| D012 | 160174950 | Legionella antigen qualitative (urine) |
| D012 | 160175450 | Helicobacter pylori antigen qualitative |
| D012 | 160177050 | Chlamydophila pneumoniae IgM antibody |
| D012 | 160177150 | Streptococcus pneumoniae antigen qualitative (urine) |
| D012 | 160188850 | Pneumococcal cell wall antigen, qualitative |
| D012 | 160188950 | Herpes simplex virus antigen qualitative (corneal) |
| D012 | 160193510 | ASO semi quantitative |
| D012 | 160193610 | ASO assay |
| D012 | 160193710 | Toxoplasma antibody qualitative |
| D012 | 160193810 | ASK semi quantitative |
| D012 | 160193910 | Mycoplasma antibody semi quantitative |
| D012 | 160194010 | Treponema pallidum antibody quantitative |
| D012 | 160194110 | Rotaviral antigen quantitative (stool) |
| D012 | 160194210 | Bordetella pertussis antibody qualitative |
| D012 | 160194310 | HTLV-1 antibody qualitative |
| D012 | 160194410 | Mycobacterial antibody quantitative |
| D012 | 160194510 | HIV-1 and -2 antibody semi quantitative |
| D012 | 160194610 | HIV-1 and -2 antibody assay |
| D012 | 160194710 | Simultaneous measurement of HIV-1 and -2 antigen/antibody, qualitative |
| D012 | 160194810 | Simultaneous measurement of HIV-1 and -2 antigen/antibody, quantitative |
| D012 | 160194910 | Candida antigen semi quantitative |
| D012 | 160195010 | Candida antigen quantitative |
| D012 | 160195110 | Norovirus antigen, qualitative |
| D012 | 160195210 | Cryptococcus antigen semi quantitative |
| D012 | 160195310 | Tsutsugamushi antibody semi quantitative |
| D012 | 160201550 | Anti-Trichosporon asahii antibody |
| D012 | 160201850 | Streptococcus pneumoniae antigen qualitative (spinal fluid) |
| D012 | 160201950 | Herpes simplex virus antigen qualitative (genital organ) |
| D012 | 160202050 | Mycoplasma antigen, qualitative (immunochromatography method) |
| D012 | 160202550 | Human metapneumovirus antigen qualitative |
| D012 | 160203010 | STS semi quantitative |
| D012 | 160203110 | FTA-ABS semi quantitative |
| D012 | 160204610 | Virus antibody titer (varicella-zoster virus) by globulin class |
| D012 | 160205550 | Dengue virus antigen qualitative |
| D012 | 160208950 | Simultaneous measurement of dengue virus antigen/antibody, qualitative |
| D012 | 160223550 | SARS-CoV-2 antigen detection |
| D012 | 160224250 | SARS-CoV-2 antigen detection (quantitative) |
| D012 | 160225550 | HIV-1 specific antibody and HIV-2 specific antibody |
| D012 | 160226450 | SARS-CoV-2/Influenza viral antigen simultaneous detection |
| D012 | 160229850 | SARS-CoV-2 antigen detection (qualitative) |
| D012 | 160229950 | SARS-CoV-2 antigen detection (quantitative) |
| D012 | 160230050 | SARS-CoV-2/Influenza viral antigen simultaneous detection (qualitative) |
| D012 | 160230550 | Dermatophyte antigen qualitative |
| D013 | 160046810 | HBsAg (qualitative/semi-quantitative) |
| D013 | 160047410 | HBs antibody semi quantitative |
| D013 | 160049210 | HBs antigen |
| D013 | 160049510 | HBs antibody |
| D013 | 160050010 | HBe antigen |
| D013 | 160050110 | HBe antibody |
| D013 | 160118510 | HCV qualitative/assay |
| D013 | 160120710 | HBc antibody titer qualitative/semiquantitative |
| D013 | 160120810 | HA antibody |
| D013 | 160120910 | HA-IgM antibody |
| D013 | 160121010 | HBc-IgM antibody |
| D013 | 160153250 | HCV specific antibody titer |
| D013 | 160153850 | HCV core antibody |
| D013 | 160154550 | HCV structural protein and nonstructural protein antibody titer |
| D013 | 160162450 | HCV, determination by serogroup |
| D013 | 160167750 | HCV core protein |
| D013 | 160182050 | HBcrAg |
| D013 | 160189050 | HBV genotype determination |
| D013 | 160189450 | HE-IgA antibody qualitative |
| D013 | 160195410 | HBs antibody qualitative |
| D013 | 160195510 | HCV structural protein and nonstructural protein antibody semiquantitative |
| D014 | 160052710 | Cold agglutinin |
| D015 | 160054610 | CRP qualitative |
| D015 | 160054710 | CRP |
| D015 | 160225950 | Interferon (IFN)-lambda 3 |
| D015 | 160227050 | TARC (COVID-19) |
| D017 | 160057850 | S-thermal insulation device use amoeba M |
| D017 | 160185570 | Collected cells smear add-on fee |
| D018 | 160058210 | Bacteriologic culture identification test (oral) |
| D018 | 160058310 | Bacteriologic culture identification test (gastrointestinal) |
| D018 | 160058410 | Bacteriologic culture identification test (urinary) |
| D018 | 160058610 | Bacteriologic culture identification test (blood) |
| D018 | 160058710 | Bacteriologic culture identification test (other) |
| D018 | 160058810 | Bacteriologic culture identification test (simple culture) |
| D018 | 160058970 | Anaerobic culturing add-on fee (bacteriologic culture identification test) |
| D018 | 160144410 | Bacteriologic culture identification test (airway) |
| D018 | 160144510 | Bacteriologic culture identification test (respiratory) |
| D018 | 160144610 | Bacteriologic culture identification test (reproductive organ) |
| D018 | 160144710 | Bacteriologic culture identification test (puncture fluid) |
| D018 | 160212670 | Mass spectrometer add-on fee (bacterial culture identification test) |
| D019 | 160146210 | Strain drug susceptibility test (one species) |
| D019 | 160146310 | Strain drug susceptibility test (2 species) |
| D019 | 160146410 | Strain drug susceptibility (more than 3 species) |
| D019 | 160217910 | Drug tolerance organism detection |
| D019 | 160218010 | Concurrent antibacterial agent effect screening |
| D019-2 | 160164450 | Drug susceptibility testing for yeast-like fungi |
| D020 | 160059210 | Mycobacterial isolation culture test (liquid nutrient medium) (oxygen sensitivity fluorescence sensor) |
| D020 | 160169910 | Mycobacterial isolation culture test (other) |
| D020 | 160172250 | Mycobacterial isolation culture test (liquid medium method) (carbon-dioxide sensor) |
| D020 | 160172350 | Mycobacterial isolation culture test (liquid medium method) (redox coloring pigment) |
| D021 | 160059410 | Mycobacterial identification |
| D022 | 160059610 | Mycobacterial drug sensitivity test |
| D023 | 160142150 | MAC nucleic acid detection |
| D023 | 160152150 | Mycobacterial nucleic acid identification |
| D023 | 160156650 | Staphylococcal methicillin resistance gene detection |
| D023 | 160157850 | Mycobacterium tuberculosis complex nucleic acid detection |
| D023 | 160158450 | HCV nucleic acid quantitative |
| D023 | 160158650 | Chlamydia trachomatis nucleic acid detection |
| D023 | 160159750 | HCV nucleic acid detection |
| D023 | 160160350 | HBV nucleic acid quantitative |
| D023 | 160163650 | HIV-1 nucleic acid, assay |
| D023 | 160164150 | Gonococcus nucleic acid detection |
| D023 | 160170070 | Concentration pretreatment add-on fee (HIV-1 nucleic acid, assay) |
| D023 | 160173350 | Detection of nucleic acids in bacteria (leukocytes) (1 strain) |
| D023 | 160174550 | Mycobacterium tuberculosis complex rifampicin resistance gene detection |
| D023 | 160175150 | Detection of precore mutation and core promoter mutation in HBV nucleic acid |
| D023 | 160175550 | SARS corona virus nucleic acid detection |
| D023 | 160177650 | Gonococcus and chlamydia trachomatis nucleic acid simultaneous detection |
| D023 | 160178710 | HIV genotype drug resistance |
| D023 | 160185610 | Detection of HPV nucleic acid |
| D023 | 160189150 | HPV genotype determination |
| D023 | 160189550 | Legionella nucleic acid detection |
| D023 | 160189650 | Mycoplasma nucleic acid detection |
| D023 | 160198010 | Influenza nucleic acid detection |
| D023 | 160201450 | Detection of HPV nucleic acid (simple genotype determination) |
| D023 | 160206710 | Detection of HTLV-1 nucleic acid |
| D023 | 160208710 | Herpes simplex virus/varicella-zoster virus nucleic acid assay |
| D023 | 160209450 | Bordetella pertussis nucleic acid detection |
| D023 | 160210150 | Bacterial nucleic acid/drug resistance gene simultaneous detection |
| D023 | 160210450 | Cytomegalovirus nucleic acid detection |
| D023 | 160212710 | EB virus nucleic acid quantification |
| D023 | 160212870 | Rapid microbial nucleic acid identification/quantitative test add-on fee |
| D023 | 160216850 | Virus/bacterial nucleic acid multi-item simultaneous detection |
| D023 | 160223350 | SARS-CoV-2 nucleic acid detection (outsourcing labs) |
| D023 | 160223450 | SARS-CoV-2 nucleic acid detection (other than outsourcing labs) |
| D023 | 160224050 | Virus/bacterial nucleic acid multi-item simultaneous detection (outsourcing labs) |
| D023 | 160224150 | Virus/bacterial nucleic acid multi-item simultaneous detection (other than outsourcing labs) |
| D023 | 160224550 | Cytomegalovirus nucleic acid assay |
| D023 | 160224850 | SARS-CoV-2/Influenza nucleic acid simultaneous detection (other than outsourcing labs) |
| D023 | 160229450 | SARS-CoV-2 nucleic acid detection (outsourcing labs) |
| D023 | 160229550 | SARS-CoV-2 nucleic acid detection (other than outsourcing labs) |
| D023 | 160229750 | SARS-CoV-2/Influenza nucleic acid simultaneous detection (other than outsourcing labs) |
| D023 | 160234950 | Virus/bacterial nucleic acid multi-item simultaneous detection (spinal fluid) |
| D023-2 | 160162650 | Escherichia coli verotoxin, qualitative |
| D023-2 | 160172850 | UBT |
| D023-2 | 160173050 | PBP2', qualitative |
| D023-2 | 160216150 | Clostridioides difficile toxin B gene detection |
| D025 | 160145410 | Basic specimen test fee (less than four weeks) |
| D025 | 160165310 | Basic specimen test fee (more than four weeks) |
| D026 | 160061810 | Hematologic test judgment fee |
| D026 | 160062110 | Immunological test judgment fee |
| D026 | 160062210 | Microbiological test judgment fee |
| D406-2 | 160097550 | Peritonsillar abscess test puncture (unilateral) |

**Supplementary Table 3.** List of antibacterials

| Minocycline hydrochloride | Kanamycin sulfate |
| --- | --- |
| Chloramphenicol sodium succinate | Gentamicin sulfate |
| Aspoxicillin hydrate | Dibekacin sulfate |
| Ampicillin sodium | Tobramycin |
| Ampicillin sodium/cloxacillin sodium hydrate | Aztreonam |
| Piperacillin sodium | Imipenem hydrate/cilastatin sodium |
| Ampicillin sodium/sulbactam sodium | Doripenem hydrate |
| Tazobactam sodium/piperacillin sodium | Panipenem/betamipron |
| Cefazolin sodium | Biapenem |
| Cefotiam hydrochloride | Meropenem hydrate |
| Cefminox sodium hydrate | Teicoplanin |
| Cefmetazole sodium | Vancomycin hydrochloride |
| Flomoxef sodium | Daptomycin |
| Cefotaxime sodium | Tedizolid phosphate |
| Cefoperazone sodium | Fosfomycin sodium |
| Cefoperazone sodium/sulbactam sodium | Linezolid |
| Ceftazidime hydrate | Amphotericin B |
| Ceftriaxone sodium hydrate | Itraconazole |
| Latamoxef sodium | Fluconazole |
| Cefepime dihydrochloride hydrate | Fosfluconazole |
| Cefozopran hydrochloride | Voriconazole |
| Cefpirome sulfate | Posaconazole |
| Ceftolozane sulfate/tazobactam sodium | Caspofungin acetate |
| Sulfamethoxazole/trimethoprim | Micafungin sodium |
| Azithromycin hydrate | Streptomycin sulfate |
| Erythromycin lactobionate | Isoniazid |
| Clindamycin phosphate | Aciclovir |
| Lincomycin hydrochloride hydrate | Ganciclovir |
| Ciprofloxacin | Vidarabine |
| Pazufloxacin mesilate | Foscarnet sodium hydrate |
| Lascufloxacin hydrochloride | Letermovir |
| Levofloxacin hydrate | Peramivir hydrate |
| Benzylpenicillin potassium | Remdesivir |
| Amikacin sulfate | Peginterferon alfa-2a (genetical recombination) |
| Arbekacin sulfate |  |
| Isepamicin sulfate |  |

All the antibacterial drugs listed are intravenous injections or infusions

**Supplementary Table 4.** Baseline characteristics (full data)

|  | | Unadjusted | | | Adjusted by SMRW | | |
| --- | --- | --- | --- | --- | --- | --- | --- |
|  |  | Exposure group  (*N* = 321) | Control group (*N* = 319) | Standardized difference | Exposure group  (*N* *=* 321.0) | Control group (*N* = 298.3) | Standardized difference |
| Sex | Male | 1 (0.3) | 0 (0.0) | 0.079 | 1.0 (0.3) | 0.0 (0.0) | 0.079 |
|  | Female | 320 (99.7) | 319 (100.0) | −0.079 | 320.0 (99.7) | 298.3 (100.0) | −0.079 |
| Age (years) | Mean ± SD | 59.2 ± 12.6 | 59.2 ± 12.7 | −0.001 | 59.2 ± 12.6 | 61.1 ± 11.2 | −0.162 |
|  | 18 to <75 | 286 (89.1) | 282 (88.4) | 0.022 | 286.0 (89.1) | 266.4 (89.3) | −0.007 |
|  | ≥75 | 35 (10.9) | 37 (11.6) | −0.022 | 35.0 (10.9) | 31.9 (10.7) | 0.007 |
| Breast cancer duration (months) | Mean ± SD | 27.58 ± 24.58 | 22.18 ± 28.76 | 0.202 | 27.58 ± 24.58 | 33.54 ± 28.74 | −0.223 |
|  | <3 | 43 (13.4) | 72 (22.6) | −0.241 | 43.0 (13.4) | 42.9 (14.4) | −0.028 |
|  | 3 to <6 | 15 (4.7) | 69 (21.6) | −0.518 | 15.0 (4.7) | 16.8 (5.6) | −0.044 |
|  | 6 to <12 | 37 (11.5) | 55 (17.2) | −0.163 | 37.0 (11.5) | 36.0 (12.1) | −0.017 |
|  | 12 to <24 | 79 (24.6) | 21 (6.6) | 0.513 | 79.0 (24.6) | 47.3 (15.9) | 0.219 |
|  | ≥24 | 147 (45.8) | 102 (32.0) | 0.286 | 147.0 (45.8) | 155.3 (52.1) | −0.126 |
| COVID-19 season | Nov 27, 2019, to Jan 14, 2020 | 22 (6.9) | 20 (6.3) | 0.024 | 22.0 (6.9) | 19.1 (6.4) | 0.018 |
|  | Jan 15 to Jul 14, 2020 | 124 (38.6) | 83 (26.0) | 0.272 | 124.0 (38.6) | 117.3 (39.3) | −0.014 |
|  | Jul 15, 2020, to Jan 14, 2021 | 59 (18.4) | 76 (23.8) | −0.134 | 59.0 (18.4) | 54.2 (18.2) | 0.005 |
|  | Jan 15 to Jul 14, 2021 | 84 (26.2) | 83 (26.0) | 0.003 | 84.0 (26.2) | 80.5 (27.0) | −0.019 |
|  | Jul 15, 2021, to Jan 14, 2022 | 18 (5.6) | 32 (10.0) | −0.165 | 18.0 (5.6) | 15.8 (5.3) | 0.014 |
|  | Jan 15 to May 31, 2022 | 14 (4.4) | 25 (7.8) | −0.146 | 14.0 (4.4) | 11.4 (3.8) | 0.027 |
| Renal impairment | No (≥90 days) | 254 (79.1) | 222 (69.6) | 0.220 | 254.0 (79.1) | 237.3 (79.6) | −0.011 |
|  | No (<90 days) | 36 (11.2) | 60 (18.8) | −0.214 | 36.0 (11.2) | 34.3 (11.5) | −0.009 |
|  | Yes | 31 (9.7) | 37 (11.6) | −0.063 | 31.0 (9.7) | 26.6 (8.9) | 0.025 |
| Hepatic impairment | No (≥90 days) | 192 (59.8) | 174 (54.5) | 0.107 | 192.0 (59.8) | 170.6 (57.2) | 0.053 |
|  | No (<90 days) | 29 (9.0) | 54 (16.9) | −0.236 | 29.0 (9.0) | 29.1 (9.8) | −0.025 |
|  | Yes | 100 (31.2) | 91 (28.5) | 0.057 | 100.0 (31.2) | 98.6 (33.1) | −0.041 |
| Lymph node metastasis | No (≥90 days) | 127 (39.6) | 178 (55.8) | −0.329 | 127.0 (39.6) | 138.7 (46.5) | −0.140 |
|  | No (<90 days) | 27 (8.4) | 55 (17.2) | −0.266 | 27.0 (8.4) | 25.0 (8.4) | 0.001 |
|  | Yes | 167 (52.0) | 86 (27.0) | 0.530 | 167.0 (52.0) | 134.5 (45.1) | 0.139 |
| Diabetes | No (≥90 days) | 242 (75.4) | 214 (67.1) | 0.184 | 242.0 (75.4) | 226.2 (75.8) | −0.011 |
|  | No (<90 days) | 34 (10.6) | 56 (17.6) | −0.201 | 34.0 (10.6) | 32.0 (10.7) | −0.004 |
|  | Yes | 45 (14.0) | 49 (15.4) | −0.038 | 45.0 (14.0) | 40.1 (13.4) | 0.017 |
| CCI score | Mean ± SD | 7.5 ± 2.8 | 5.8 ± 3.7 | 0.535 | 7.5 ± 2.8 | 7.8 ± 2.8 | −0.090 |
|  | <7 | 62 (19.3) | 151 (47.3) | −0.623 | 62.0 (19.3) | 53.1 (17.8) | 0.039 |
|  | ≥7 | 259 (80.7) | 168 (52.7) | 0.623 | 259.0 (80.7) | 245.2 (82.2) | −0.039 |
| Prior breast cancer surgery | No (≥90 days) | 277 (86.3) | 237 (74.3) | 0.305 | 277.0 (86.3) | 258.6 (86.7) | −0.011 |
|  | No (<90 days) | 38 (11.8) | 62 (19.4) | −0.210 | 38.0 (11.8) | 35.5 (11.9) | −0.002 |
|  | Yes | 6 (1.9) | 20 (6.3) | −0.224 | 6.0 (1.9) | 4.2 (1.4) | 0.037 |
| Prior non-breast cancer surgery | No (≥90 days) | 99 (30.8) | 111 (34.8) | −0.084 | 99.0 (30.8) | 90.1 (30.2) | 0.014 |
|  | No (< 90 days) | 29 (9.0) | 50 (15.7) | −0.203 | 29.0 (9.0) | 25.4 (8.5) | 0.018 |
|  | Yes | 193 (60.1) | 158 (49.5) | 0.214 | 193.0 (60.1) | 182.7 (61.3) | −0.023 |
| Prior radiotherapy | No (≥90 days) | 150 (46.7) | 209 (65.5) | −0.386 | 150.0 (46.7) | 160.1 (53.7) | −0.140 |
|  | No (<90 days) | 36 (11.2) | 62 (19.4) | −0.230 | 36.0 (11.2) | 35.5 (11.9) | −0.022 |
|  | Yes | 135 (42.1) | 48 (15.0) | 0.627 | 135.0 (42.1) | 102.6 (34.4) | 0.158 |
| Prior anticancer treatment | No (≥90 days) | 24 (7.5) | 23 (7.2) | 0.010 | 24.0 (7.5) | 13.0 (4.4) | 0.133 |
|  | No (<90 days) | 29 (9.0) | 56 (17.6) | −0.253 | 29.0 (9.0) | 33.7 (11.3) | −0.075 |
|  | Yes | 268 (83.5) | 240 (75.2) | 0.205 | 268.0 (83.5) | 251.5 (84.3) | −0.023 |
| Steroid use | No (≥90 days) | 163 (50.8) | 82 (25.7) | 0.534 | 163.0 (50.8) | 129.7 (43.5) | 0.147 |
|  | No (<90 days) | 29 (9.0) | 58 (18.2) | −0.269 | 29.0 (9.0) | 34.8 (11.7) | −0.086 |
|  | Yes | 129 (40.2) | 179 (56.1) | −0.323 | 129.0 (40.2) | 133.8 (44.9) | −0.095 |

Exposure group: atezolizumab plus nab-paclitaxel. Control group: nab-paclitaxel alone. Data are presented as *n* (%) unless otherwise specified. No (≥90 days) and no (<90 days) were categorized based on the presence of medical treatment before the baseline period

*CCI* Charlson Comorbidity Index, *COVID-19* coronavirus disease 2019, *SD* standard deviation, *SMRW* standardized mortality/morbidity ratio weighting

**Supplementary Table 5**. Clinical events related to severe infections during the observation period (original definition)

|  | | Unadjusted | | | Adjusted by SMRW | | |
| --- | --- | --- | --- | --- | --- | --- | --- |
|  |  | Exposure group  (*N* = 321) | Control group (*N* = 319) | Standardized difference | Exposure group  (*N* = 321.0) | Control group (*N* = 298.3) | Standardized difference |
| Number of hospitalizations | Mean ± SD | 0.6 ± 1.2 | 0.4 ± 0.9 | 0.174 | 0.6 ± 1.2 | 0.4 ± 0.8 | 0.156 |
| Number of surgeries | Mean ± SD | 0.1 ± 0.4 | 0.0 ± 0.2 | 0.283 | 0.1 ± 0.4 | 0.1 ± 0.3 | 0.2 |
| Number of radiotherapies | Mean ± SD | 0.9 ± 3.2 | 0.6 ± 2.6 | 0.096 | 0.9 ± 3.2 | 0.8 ± 3.4 | 0.015 |
| Steroid use | No | 118 (36.8) | 69 (21.6) | 0.337 | 118.0 (36.8) | 66.7 (22.4) | 0.32 |
|  | Yes | 203 (63.2) | 250 (78.4) | −0.337 | 203.0 (63.2) | 231.6 (77.6) | −0.32 |

Exposure group: atezolizumab plus nab-paclitaxel. Control group: nab-paclitaxel alone

*SD* standard deviation, *SMRW* standardized mortality/morbidity ratio weighting

**Supplementary Table 6**. Most commonly prescribed antibacterial drugs (Outcome Definition 2): additional analysis

|  | Exposure group (*N* = 51) | Control group (*N* = 26) |
| --- | --- | --- |
| Antibacterial drugs prescribed during the observation period, *n* (%) | | |
| Sulbacillin for Intravenous Injection 1.5 g | 4 (7.8) | 3 (11.5) |
| Cefazolin Sodium for Intravenous Infusion 1 g Bag with Otsuka Physiological Saline 100 mL | 9 (17.6) | 1 (3.8) |
| Cefazolin Sodium for Injection 1 g “Nichi-Iko Pharmaceutical” | 5 (9.8) | 3 (11.5) |
| Ceftriaxone Sodium for Intravenous Injection 1 g “Nichi-Iko Pharmaceutical” | 3 (5.9) | 4 (15.4) |
| Meropenem for Intravenous Infusion 0.5 g “Meiji” 500 mg | 3 (5.9) | 1 (3.8) |
| Cefepime Dihydrochloride for Intravenous Injection 1 g “Sandoz” | 0 (0.0) | 2 (7.7) |
| Tazopipe Combination for Intravenous Injection 4.5 “Meiji” 4.5 g | 7 (13.7) | 4 (15.4) |
| Maxipime for Injection 1 g | 1 (2.0) | 2 (7.7) |
| Antibacterial drugs prescribed at the onset of severe infections, *n* (%) | | |
| Sulbacillin for Intravenous Injection 1.5 g | 4 (7.8) | 3 (11.5) |
| Cefazolin Sodium for Intravenous Infusion 1 g Bag with Otsuka Physiological Saline 100 mL | 7 (13.7) | 1 (3.8) |
| Cefazolin Sodium for Injection 1 g “Nichi-Iko Pharmaceutical” | 5 (9.8) | 2 (7.7) |
| Ceftriaxone Sodium for Intravenous Injection 1 g “Nichi-Iko Pharmaceutical” | 2 (3.9) | 4 (15.4) |
| Cefepime Dihydrochloride for Intravenous Injection 1 g “Sandoz” | 0 (0.0) | 2 (7.7) |
| Tazopipe Combination for Intravenous Injection 4.5 “Meiji” 4.5 g | 6 (11.8) | 4 (15.4) |
| Maxipime for Injection 1 g | 0 (0.0) | 2 (7.7) |

Exposure group: atezolizumab plus nab-paclitaxel. Control group: nab-paclitaxel alone Antibacterial drug products that were prescribed to ≥5% of patients in either group are listed

**Supplementary Fig. 1**. Definitions of severe infections


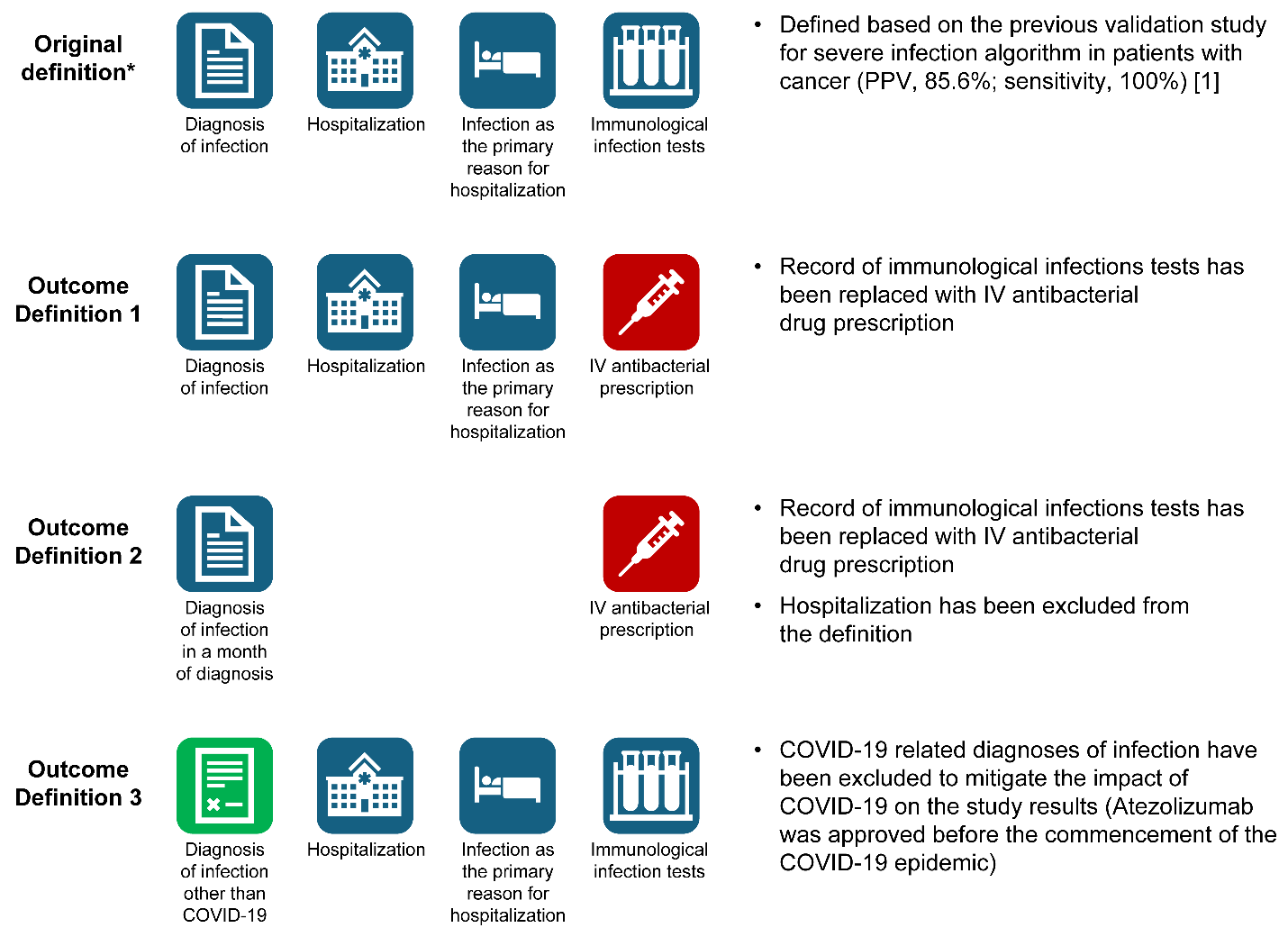


*In patients with breast cancer, hospitalizations due to noninfectious diseases may have occurred more frequently than in the previous validation study [1]. Patients included in the current postmarketing study may have undergone infection tests as part of pre-hospitalization examinations even if they had no signs or symptoms of infections

*COVID-19* coronavirus disease 2019, *IV* intravenous, *PPV* positive predictive value

[1] Nishikawa A, Yoshinaga E, Nakamura M et al (2022) Validation study of algorithms to identify malignant tumors and serious infections in a Japanese administrative healthcare database. Ann Clin Epidemiol 4(1):20–31. <https://doi.org/10.37737/ace.22004>

**Supplementary Fig. 2**. Propensity score distribution (a) before and (b) after weighting


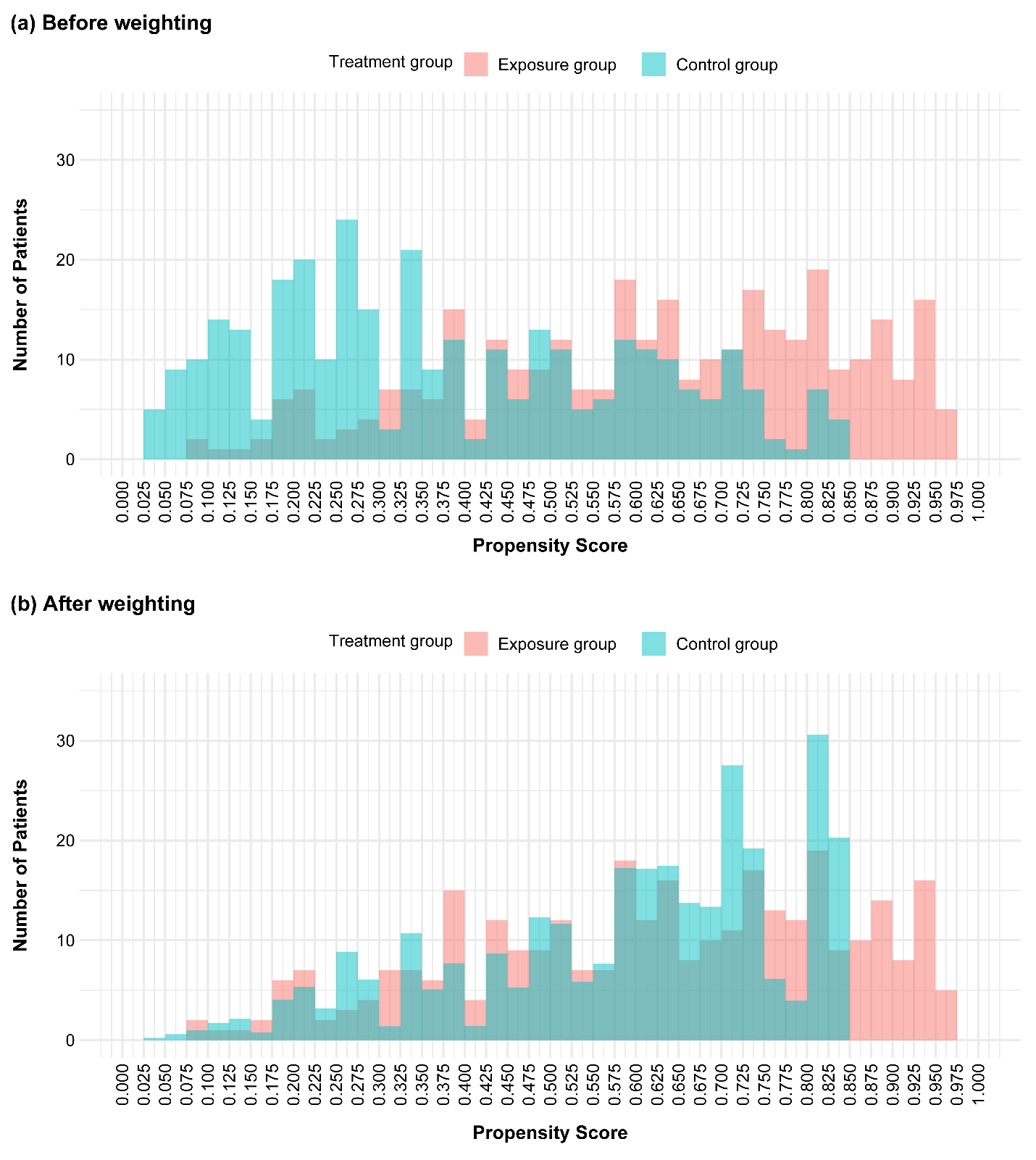


Exposure group: atezolizumab plus nab-paclitaxel. Control group: nab-paclitaxel alone

**Supplementary Fig. 3**. Time to the onset of severe infections using (a) original definition and (b) Outcome Definition 2 (sensitivity analysis)


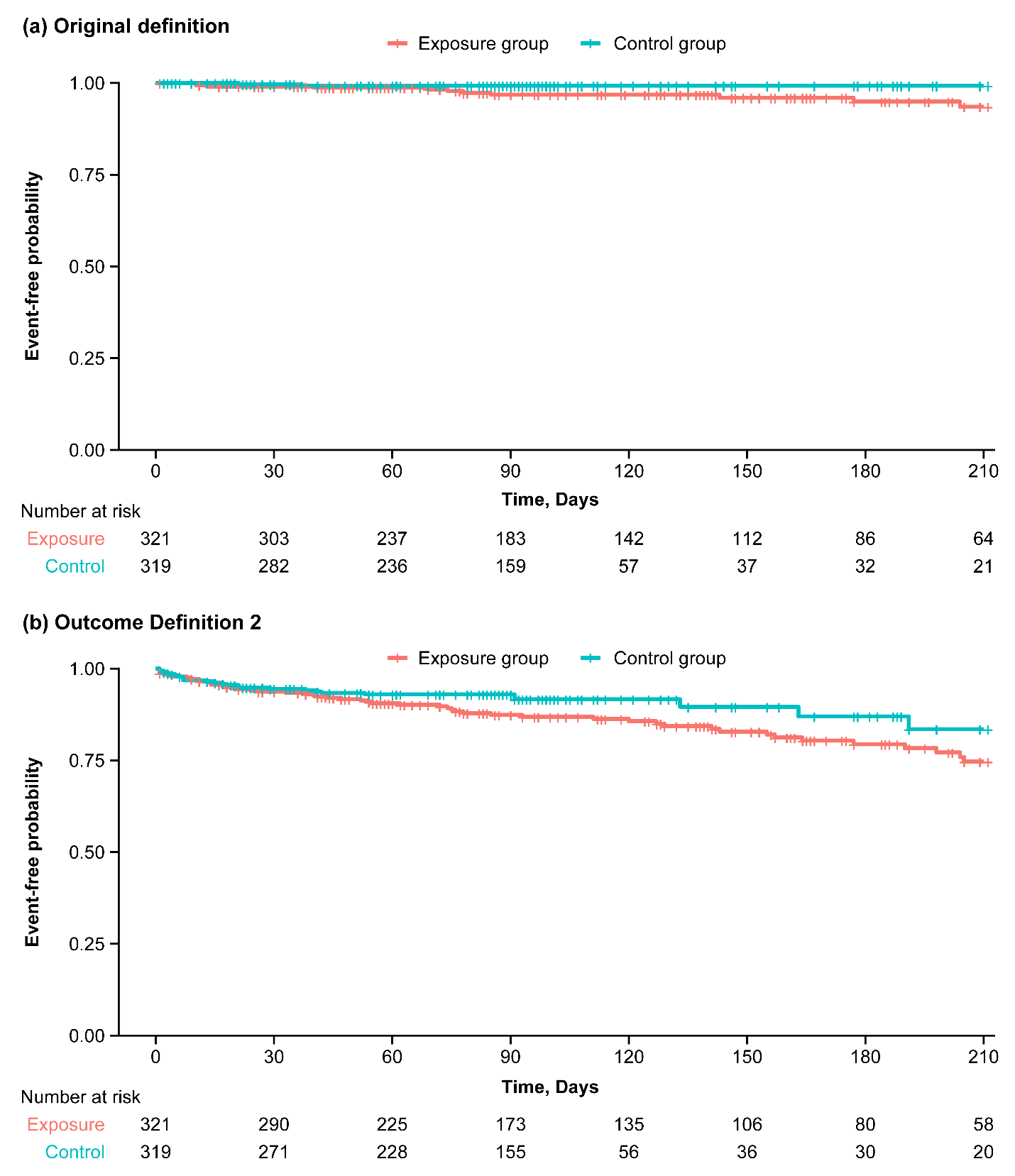


Exposure group: atezolizumab plus nab-paclitaxel. Control group: nab-paclitaxel alone

**Supplementary Fig. 4**. Time to the onset of severe infections using (a) Outcome Definition 1 (adjusted for SMRW) and (b) Outcome Definition 3 (adjusted for SMRW)


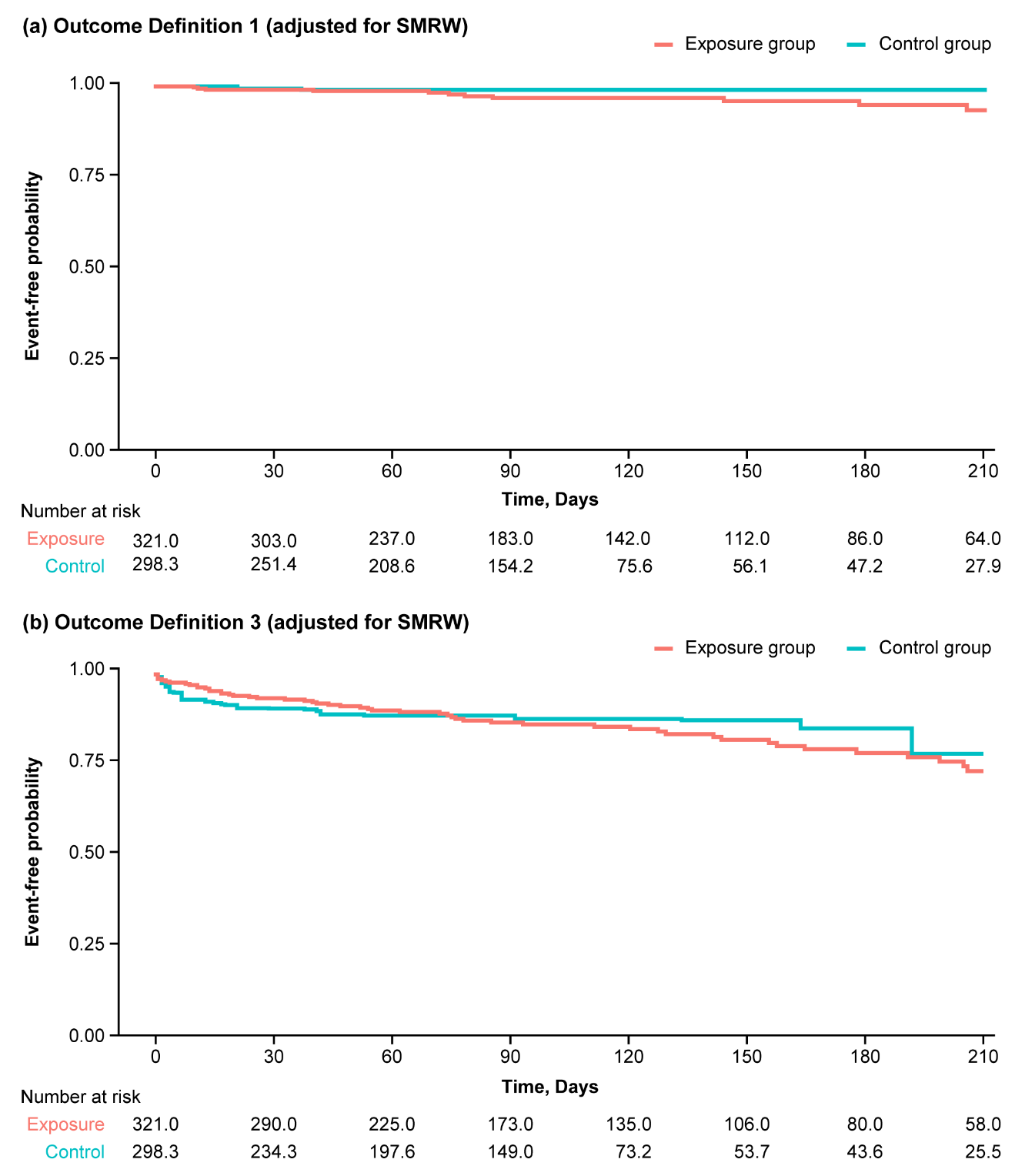


Exposure group: atezolizumab plus nab-paclitaxel. Control group: nab-paclitaxel alone

*SMRW* standardized mortality/morbidity ratio weighting
